# The Effect of Maternal Healthcare on the Probability of Child Survival in Azerbaijan

**DOI:** 10.1155/2014/317052

**Published:** 2014-07-10

**Authors:** Nazim Habibov, Lida Fan

**Affiliations:** ^1^School of Social Work, University of Windsor, Windsor, ON, Canada N9B 3P4; ^2^School of Social Work, Lakehead University, Thunder Bay, ON, Canada P7B 5E1

## Abstract

This study assesses the effects of maternal healthcare on child survival by using nonrandomized data from a cross-sectional survey in Azerbaijan. Using 2SLS and simultaneous equation bivariate probit models, we estimate the effects of delivering in healthcare facility on probability of child survival taking into account self-selection into the treatment. For women who delivered at healthcare facilities, the probability of child survival increases by approximately 18%. Furthermore, if every woman had the opportunity to deliver in healthcare facility, then the probability of child survival in Azerbaijan as a whole would have increased by approximately 16%.

## 1. Introduction

Poor child outcomes are usually associated with underutilization of maternal healthcare [1–3]. Given unusually high mortality rates in countries of Central Asia and Caucasus, poor child outcomes and maternal healthcare should become important topics for research. Nevertheless, there are a very few studies on these topics in the region. The available studies can be divided into two broader groups. The first group explored determinants of child mortality [4, 5]. The second group explored determinants of maternal healthcare utilization [2, 6–9]. Although these studies have important contributions, their main limitation is that the most important question on whether healthcare has an effect on the reduction of child mortality is overlooked. However, designing and implementing effective health policy require concrete information on the effectiveness of the existing maternal healthcare.

The contribution of the presented study is that it attempts to fill the gap in the existing literature by quantifying the direct effect of delivery in healthcare facility on probability of child survival. The robust evaluation of program effect on population usually involves randomized control trials (RCT). In many cases, including evaluation of maternal healthcare, conducting a RCT is not possible from an ethical perspective, withholding vital service, and from technical perspective, lack of money and time required to conduct a countrywide RCT. To overcome these difficulties, we assess the effect of healthcare and homecare on child survival by using quasiexperimental evaluation of nonrandomized data from a cross-sectional survey. In this way, this study contributes to the recent discussion on appropriate methods for the evaluation of effect of healthcare programs when RCT is not feasible [10–12].

Azerbaijan, a low-income transitional country on Caucasus, is an interesting setting for examining the above-mentioned issues for several reasons. First, Azerbaijan has the highest infant mortality rate and one of the highest proportions of child deliveries outside of healthcare facilities even compared with other transitional countries in the region [13]. Second, by studying Azerbaijan, we benefit from recently available 2006 Azerbaijan Demographics and Health Survey that contains high-quality nationally representative data on the issues of our interest. Third, there is a current theoretical debate on the actual effectiveness of maternal healthcare in transitional countries. On the one hand, maternal healthcare is universal, officially free of charge, fully funded, and operated by the government. It has an extensive network of facilities which is adequately staffed with qualified personnel. Hence, a fairly strong positive impact on child survival could be expected and some authors underscore the importance of maternal healthcare utilization in transitional countries to improve child outcomes [6–9]. On the other hand, the system is characterized by chronic underfunding, lack of drugs and supplies, dilapidated facilities, lack of systematic and effective treatments, and high levels of unofficial out-of-pocket expenditures for personnel [14–16]. Hence, no or only weak impact on the child survival could be expected. Therefore, by focusing on Azerbaijan, a transitional country, this study provides necessary empirical evidence which will contribute to the current theoretical debate on the effectiveness of maternal healthcare in transitional countries.

## 2. Materials and Methods

### 2.1. Conceptual Framework

We are guided by Mosley and Chen's [18] framework for studying the determinants of child survival. According to the framework, socioeconomic determinants at individual (e.g., women's education), household (e.g., household income), and community (e.g., healthcare input) levels affect a total of 14 proximate determinants of mortality which are grouped into several categories, namely, maternal factors, environmental contamination, nutrient deficiency, and personal illness control. However, the model has a few limitations for applied research. Some proximate determinants, for instance, environmental contamination, are notoriously difficult to define and measure adequately, especially in population based surveys [19]. Furthermore, if a model includes all socioeconomic and all proximate determinants, then the coefficients on the socioeconomic variables should not be statistically significant given that the proximate determinants will pick up all significance by definition [20]. Consequently, we reduced the number of independent variables to women's age at birth and education, birth order, low child birthweight, household wealth, and healthcare input. As a result, we used the reduced set of independent variables which is similar to previous studies on child survival in the region [4, 5, 21, 22] and international comparative studies [23].

### 2.2. Method

We are interested in estimating effect of treatment, having child delivery at a healthcare facility, on the outcome, probability of child survival. Thus, we face a problem of self-selection—the sampled individuals who receive the treatment are different from those who do not receive it in unobservable ways which are also simultaneously correlated with outcome [10, 12]. To address the self-selection we use simultaneous equation regression that tackles the endogeneity by specifying and estimating a joint model of the treatment and outcome [24–27]. Since both treatment and outcome variable in our case are binomial, we use a simultaneous equation bivariate probit, so-called biprobit. The model consists of first and main equations. In the first equation, a dummy treatment variable is regressed on all control variables and one or more instruments. In the main equation, a dummy outcome variable is regressed on all control variables and the value of the treatment variable estimated in the first stage. Importantly, the instruments are excluded from the main equation. This statistical specification is estimated using* biprobit *command in Stata software package [28]. After biprobit was estimated, we compute the average treatment effect (ATE) and the average treatment effect on the treated (ATT) [10]. The value of the ATE indicates the expected mean effect of the treatment for a woman drawn at random from the population. By contrast, the value of ATT indicates the expected mean effect of the treatment for a woman who actually participates in the program and receives treatment. ATT permits us to evaluate the effect on women who received treatment and who can be considered as a more relevant subpopulation for the purposes of evaluating effect of a specific program. The full details of biprobit, ATE, and ATT computations can be found in Greene [29] and Wooldridge [30].

### 2.3. Data

This study uses data from the 2006 Azerbaijan Demographic and Health Survey (the AZDHS). The AZDHS is conducted by the national statistical authority, the State Statistical Committee of Azerbaijan, with technical assistance of Macro International, USA, and with financial support from USAID and UNICEF [17]. The AZDHS is a cross-sectional survey of 8,444 women aged 15 to 49 from 7,180 households. Field work was conducted from July to November 2006. The household gross response rate exceeds 90 percent. The AZDHS gathered information on demographics, educational level, household wealth, healthcare utilization, and child mortality. The AZDHS collected information about the outcome of each respondent's pregnancy for the period, whether the pregnancy ended in a live birth, a stillbirth, a miscarriage, or an induced abortion. The survey used the international definition of child mortality, under which any birth in which a child showed any sign of life such as breathing, beating of the heart, or movement of voluntary muscles is defined as a live birth. The AZDHS collected information on child mortality for births in 2001 or later, covering a period of 5 years before the date of the survey only. Among recorded 13,565 observations, about 92% of children survived between birth and their fifth birthday and about 8% died. However, our sample is further reduced since the questions about place of delivery asked only about the most recent birth delivered during the the last 5 years before the date of the survey. It means that if a women had multiple births during the last 5 years, the questions about place of delivery was asked only about the latest birth. Consequently, our final sample consists of 2,285 observations for analysis.

### 2.4. Outcome and Treatment Variables

The outcome variable of this study is child survival defined as probability to survive during 60 months or 5 years. This variable is binomial; it takes the value of 1 if the child survives 60 months and takes the value of 0 if otherwise. There are two endogenous instrumented variables of interests which denote treatment and serve to gauge healthcare input. The instrumented treatment variable is “delivery in a healthcare facility” that takes the value of 1 if the child was delivered in a healthcare facility and takes the value of 0 if otherwise. The healthcare facility is defined as a government or private hospital, maternity home, polyclinic, woman's consultation, and primary healthcare posts. Overall, from the sample of 2,285 women who answered the questions about place of delivery in the AZDHS, approximately 79% delivered babies in a healthcare facility.

### 2.5. Instrumental Variables

The instrumental variables used to estimate the endogenous treatment variables are taken from a previous study that used instrumental variables to estimate the effect of prenatal healthcare utilization on child birthweight in Azerbaijan [31]. There are two instrumental variables—“women from wealthier households” and “birth order.” The AZDHS contains a variable representing 5 quintiles of household wealth—poorest, poor, middle, richer, and richest. We create a “wealthier households” dummy variable which denotes women from richest and richer households, and this variable is used in our model 1 and model 2. Finally, “birth order” is a straightforward continuous variable denoting number of births.

### 2.6. Exogenous Variables

The exogenous variables used to explain child survival are taken from the previous studies on the determinants of child mortality conducted in the countries of the region of Caucasus and Central Asia [4, 5, 21, 22]. We have two dummy variables representing women's age: variable “age 20” indicates women aged 20 or younger at the time of delivery, while variable “age 36” indicates women aged 36 and older at the time of delivery. Dummy variable “low birthweight” indicates if a child's birthweight was 2500 grams or lower. Dummy variable “higher education” indicates women with bachelor education or higher. Previous studies reported that having delivery at age <20 and age >35 is associated with higher probability of child mortality. Likewise, previous studies reported that having low birthweight is associated with higher probability of child mortality, while having higher educational achievements is associated with lower probability of child mortality.

### 2.7. Estimation

We commence with 2SLS model because the tests for overidentifying restrictions and the adequacy of the instruments are readily available for the 2SLS but not for biprobit [11, 12, 32]. Since the number of instrumental variables exceeds the number of endogenous variables in our case, the Hansen *J* statistic is employed to evaluate overidentifying restrictions. If Hansen *J* statistics cannot reject the null hypothesis, then the selected instrumental variables are exogenous. In addition, Kleibergen-Paap LM statistic is used to test the adequacy of the instruments. If the test rejects the null hypothesis, the instruments are adequate to identify the equation. Lastly, we conduct Durbin-Wu-Hausman test for potential endogeneity. The significance of the test confirms the presence of endogeneity and suggests that estimation of equations without taking into account endogeneity will lead to biased results. All the above-described tests have been passed in all estimated models.

Next we estimate biprobit which is more relevant model due to the binary nature of outcome and treatment variables. A straightforward Wald test of endogeneity is available in biprobit. If result of the test is significantly different from zero, then biprobit should be estimated due to the presence of endogeneity. In all estimated models, the Wald tests have confirmed endogeneity. After biprobit model estimation, ATE and ATT are computed and reported.

## 3. Results

The results are reported in Table 1. In the first equation four variables are significant with predicted directions in 2SLS estimation. Having birth at the age of 20 or earlier and having a higher value of birth order are associated with lower probability of delivery in a healthcare facility, while having higher educational achievements and being from a wealthier household are associated with higher probability of delivery in a healthcare facility. Looking at the main equation in 2SLS, we can see that having a delivery in the facility improves the chances of child survival. Results of biprobit estimation are consistent with the results of the 2SLS estimation. The same four variables are significant in the first equation and with the same direction.

ATE and ATT are reported in the last two rows of the table below biprobit estimations. ATE suggests that delivering in a healthcare facility improves the chances of child survival for a woman randomly drawn from the population by approximately 16%. In comparison, ATT suggests that delivering in a healthcare facility improves the chances of child survival for women who actually participated in the program by about 18%.

## 4. Discussion and Policy Implications

In this study, we identified and then attempted to fill the important gap in the literature regarding the effectiveness of maternal healthcare in reducing under-five child mortality in the region of the Central Asia and the Caucasus. We assessed the effects of delivering in a healthcare facility on child survival by using a quasiexperimental evaluation based on nonrandomized data from a cross-sectional survey in Azerbaijan, a low-income country in transition. The empirical evidence presented in this paper allows for drawing several conclusions.

First, delivering children in healthcare facilities increases the probability of survival. Since reducing child mortality is* raison d'être* for maternal healthcare programs, such a funding could be expected. However, we were able to confirm that the effect of delivering at a healthcare facility on child survival is statistically significant on the national level. We also quantified the positive effect of such treatment. For women who delivered at healthcare facilities the probability of child survival increases by approximately 18%. Furthermore, if every woman in Azerbaijan had the opportunity to deliver in a healthcare facility, then the probability of child survival in the country would have increased by approximately 16%. These findings suggest that utilization of maternal services in transitional countries should be encouraged and promoted in spite of the limitations and deficiencies in the current maternal healthcare system.

Second, our study demonstrates that the wealth gradient is an important barrier for utilization and hence influences the child outcomes. Since maternal healthcare is officially free, the prior studies explained the effect of wealth gradient by high level of unofficial out-of-pocket expenditures for healthcare personnel, supplies, and medication [7, 33, 34]. As a result, the wealthier use healthcare facilities which the poorer cannot afford. This is in line with our finding that the wealthier deliver in healthcare facilities, while the poorer have to deliver outside of healthcare facilities. while the poorer have to deliver at home. In this context, one of the promising ways to reduce effect of wealth gradient to utilization is to introduce the benefits for pregnant women which could be linked to receipt of targeted social assistance programs [35, 36].

Third, our study demonstrates that the risk of not delivering at a healthcare facility increased for less educated women. Women with higher education are strongly associated with delivering in medical settings and hence with higher chances of child survival. Habibov [2] reported that there is no significant gender gap in the level of literacy and education in general in Azerbaijan and concluded that increase in nonacademic educational activities promoting antenatal care should be a priority. Habibov and Fan [9] confirmed these conclusions showing the example of Tajikistan, another transitional country, that having limited knowledge on matters related to sex is associated with a lower probability of maternal healthcare utilization. The authors underlined that significant effect of knowledge about sex is independent of formal educational level and it persisted even if formal educational level is controlled for. Effectiveness of communication campaigns designed to explain the benefits of maternal healthcare and encourage healthcare utilization is well documented in developing countries [37]. In addition, intensive communication campaigns aimed at encouraging healthcare utilization slowly but steadily became appreciated in some transitional countries [8]. This positive experience should be shared across the region.

Finally, the population based nationally representative surveys such as the Demographic and Health Surveys by Macro International and Living Standards Measurement Surveys by the World Bank became an important tool for measuring policy effect on health outcomes in many transitional and developing countries [23, 33, 34]. Most of these surveys include modules on healthcare utilization and childbirth outcome [2, 6–8]. Having high-quality microdata to conduct evaluation of healthcare programs is an effective way to save time, effort, and costs while providing nationally representative results. From this standpoint, our results are illustrative to empirical strategies for evaluation of nonrandomized data from cross-sectional surveys using a standard statistical software package.

## Figures and Tables

**Table 1:** The effect of delivery in healthcare facility on probability of child survival.

	2SLS model			Bivariate probit model		
	Coef.	Std. Err.	*P* > *t*	Coef.	Std. Err.	*P* > *z*
First equation: instrumented variable is delivery in health care facility; instrumental variables are wealth and birth order						
Age 20 or younger	−0.104	0.035	0.003	−0.357	0.116	0.002
Age 36 or older	0.094	0.053	0.076	0.344	0.207	0.098
Low birthweight	0.061	0.045	0.171	0.219	0.179	0.222
Higher education	0.077	0.022	0.000	0.660	0.199	0.001
Wealth	0.198	0.032	0.000	0.891	0.144	0.000
Birth order	−0.073	0.013	0.000	−0.247	0.040	0.000
Constant	0.854	0.038	0.000	1.049	0.135	0.000

Main equation: outcome variable is probability of child survival						
Delivery in healthcare facility	0.151	0.063	0.016	0.923	0.347	0.008
Age 20 or younger	0.012	0.016	0.451	0.080	0.154	0.601
Age 36 or older	−0.017	0.031	0.584	−0.136	0.247	0.582
Low birthweight	−0.020	0.019	0.288	−0.174	0.167	0.297
Higher education	−0.032	0.022	0.144	−0.164	0.199	0.410
Constant	0.843	0.051	0.000	0.969	0.319	0.002
Number of observations	2285					
*F* (5, 311)	1.31					
Prob > *F*	0.000					
Number of observations				2285		
Log pseudo-likelihood				−1450000000		
Wald *χ* ^2^ (11)				126.52		
Prob > *χ* ^2^				0.000		

Test of endogeneity						
Durbin-Wu-Hausman test *χ* ^2^ and (*P* value)	10.49 (0.001)					
*C*-statistic *χ* ^2^ and *P* value	5.647 (0.017)					
*ρ* (Rho)				−0.424		
Wald test *χ* ^2^ and *P* value				4.15 (0.041)		

Tests for overidentifying restrictions						
Hansen *J* statistic and *P* value	0.407 (0.686)					

Tests for the adequacy of instruments						
Kleibergen-Paap LM statistic and *P* value	42.93 (0.000)					

Effects of treatment						
ATE (average effect of treatment)				0.161		
ATT (average effect of treatment to the treated)				0.184		

Notes: (1) dependent variable in the first stage is healthcarefacility delivery = 1; otherwise = 0. Dependent variable in the second stage is child survival = 1; otherwise = 0.

(2) *P < 0.05, **P < 0.01, and ***P < 0.001.

(3) Results adjusted to heteroskedasticity and clustering.

(4) Data are rounded up

Source: 2006 Azerbaijan Demographic and Health Survey [17].
